# Binding of an RNA aptamer and a partial peptide of a prion protein: crucial importance of water entropy in molecular recognition

**DOI:** 10.1093/nar/gku382

**Published:** 2014-05-06

**Authors:** Tomohiko Hayashi, Hiraku Oshima, Tsukasa Mashima, Takashi Nagata, Masato Katahira, Masahiro Kinoshita

**Affiliations:** Institute of Advanced Energy, Kyoto University, Uji, Kyoto 611-0011, Japan

## Abstract

It is a central issue to elucidate the new type of molecular recognition accompanied by a global structural change of a molecule upon binding to its targets. Here we investigate the driving force for the binding of R12 (a ribonucleic acid aptamer) and P16 (a partial peptide of a prion protein) during which P16 exhibits the global structural change. We calculate changes in thermodynamic quantities upon the R12–P16 binding using a statistical-mechanical approach combined with molecular models for water which is currently best suited to studies on hydration of biomolecules. The binding is driven by a water-entropy gain originating primarily from an increase in the total volume available to the translational displacement of water molecules in the system. The energy decrease due to the gain of R12–P16 attractive (van der Waals and electrostatic) interactions is almost canceled out by the energy increase related to the loss of R12–water and P16–water attractive interactions. We can explain the general experimental result that stacking of flat moieties, hydrogen bonding and molecular-shape and electrostatic complementarities are frequently observed in the complexes. It is argued that the water-entropy gain is largely influenced by the geometric characteristics (overall shapes, sizes and detailed polyatomic structures) of the biomolecules.

## INTRODUCTION

An intrinsically disordered protein is characterized by a lack of stable tertiary structure when it is present as an isolated polypeptide chain in aqueous solution under the physiological condition. Its folding is coupled with binding to its targets in the sense that it constructs a well-defined structure only after the binding is implemented (1). An aptamer, which is a folded single-stranded nucleic acid, possesses structural plasticity and binds to its targets by changing its structure in accordance with the target structures (2). The structure of the aptamer remains unchanged in some cases, and disordered, flexible portions of its targets become structured upon binding. This type of molecular recognition accompanied by a global structural change of a molecule upon binding to its targets appears to be substantially different from that described by the ‘lock and key’ (3) or ‘induced-fit’ (4) binding model. Elucidation of these molecular-recognition mechanisms is a central issue in biophysics, biochemistry and structural biology. In our opinion, all types of molecular recognitions share the mechanism based on the same physicochemical origin and can be explained within the same theoretical framework in a unified manner. The present study provides a remarkable first step toward achieving such elucidation by considering a ribonucleic acid (RNA) aptamer (5) as an important example.

**Figure 1.**
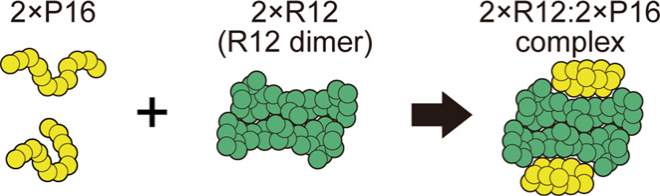
Binding of two P16s to an R12 dimer. The complex formed is denoted by ‘2×R12:2×P16’. The water molecules are omitted here.

Aptamers are RNAs and deoxyribonucleic acids originating from *in vitro* selection experiments which optimize the nucleic acids for high-affinity binding to their targets by starting from random sequence libraries (2,6,7). Up to now, many aptamers have been developed against a wide variety of targets such as small molecules (8), small interfering RNA (siRNAs) (9), peptides (10), sugars (11,12), proteins (13–17) and cells (18,19). Aptamers have many potential uses as effective therapeutics (20), drug delivery agents (21), purification agents for the therapeutic antibodies (16) and molecular probes (22). Elucidation of the molecular-recognition mechanism common in all of the aptamers presents much challenge not only from the scientific viewpoint but also for medical and technological applications. The RNA aptamer considered in the present study, r(GGAGGAGGAGGA) (R12), binds to and stabilizes a normal cellular form of a prion protein (PrP^C^) (23), which is expected to prevent prion diseases (5). R12 takes a unique quadruplex structure forming a dimer (23). In this case, two portions (P1 and P16) of the N-terminal half of PrP^C^ are disordered and flexible, and they form well-defined structures in accordance with R12 upon binding. Mashima *et al.* (5) performed experiments for binding of P16 (Gly-Gln-Trp-Asn-Lys-Pro-Ser-Lys-Pro-Lys-Thr-Asn) to R12, and we consider this fundamental binding process. In this process, two P16s bind to the R12 molecules forming the dimer, respectively, as illustrated in Figure 1. Since the upper and lower halves of the 2×R12:2×P16 complex share essentially the same structure, the theoretical investigation can be made for the constituent process in which a P16 binds to one of the R12 molecules, forming an R12:P16 complex.

**Figure 2.**
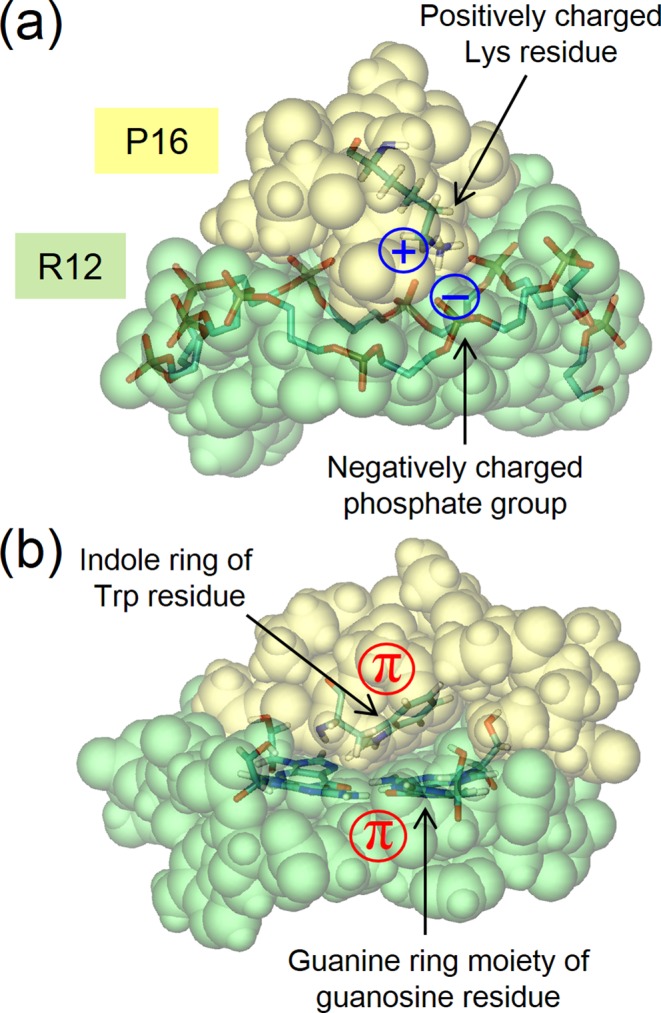
Structural images of the upper half of the R12:P16 complex. It is taken from an NMR structure (model 1) constructed as described in ‘Structure Modeling for R12:P16 Complex’. R12 and P16 are drawn on the upper and lower sides, respectively, using the space-filled model. (**a**) Contact of a phosphate group in R12 with a lysine residue in P16. The phosphate backbone (C5′-C4′-C3′-PO^4−^) and Lys8 are depicted by the licorice model. (**b**) π–π stacking of a guanine nucleotide in R12 and an indole ring of tryptophan in P16. The quadruplex tetrad plane containing G:G:G:G (G denotes a guanine nucleotide) and Trp3 are depicted by the licorice model. The water molecules are omitted here. This figure is drawn using the VMD

The clue to the molecular-recognition mechanism is the driving force for the binding. The force has been investigated primarily from the viewpoint of structural biology on the basis of the three-dimensional (3D) structure of the aptamer–target complex. A prevailing view is that an aptamer binds to its targets via aptamer–target electrostatic attractive interactions due to the contact of groups with positive and negative charges, that is, the electrostatic complementarity. Actually, in several aptamer–target complexes, negatively charged phosphate backbones in the aptamer are in contact with positively charged moieties of the targets. For example, Toggle-25t (an RNA aptamer) binds to the positively charged arginine-rich surface of thrombin (15). In the R12:P16 complex, the contact of phosphate groups of R12 with lysine residues of P16 is observed as illustrated in Figure 2 (5) (Figures 2, 4 and 5 are drawn using the Visual Molecular Dynamics (VMD)(24)). However, there is an experimental result that raises a doubt with respect to this view: in the complex of Apt8 (an RNA aptamer) binding to the Fc fragment of Human IgG1 (hFc1), the aptamer-bound area of hFc1 consists of a less-positively charged surface (16,25). In the case of the R12–P16 binding, a π–π stacking interaction between a guanine nucleotide in R12 and an indole ring of tryptophan in P16 (see Figure 2) has been proposed as another important driving force (5). Taken together, the structural data have shown that precise stacking of flat moieties, specific hydrogen bonding and electrostatic complementarity frequently occur in the complexes (2).

**Figure 3.**
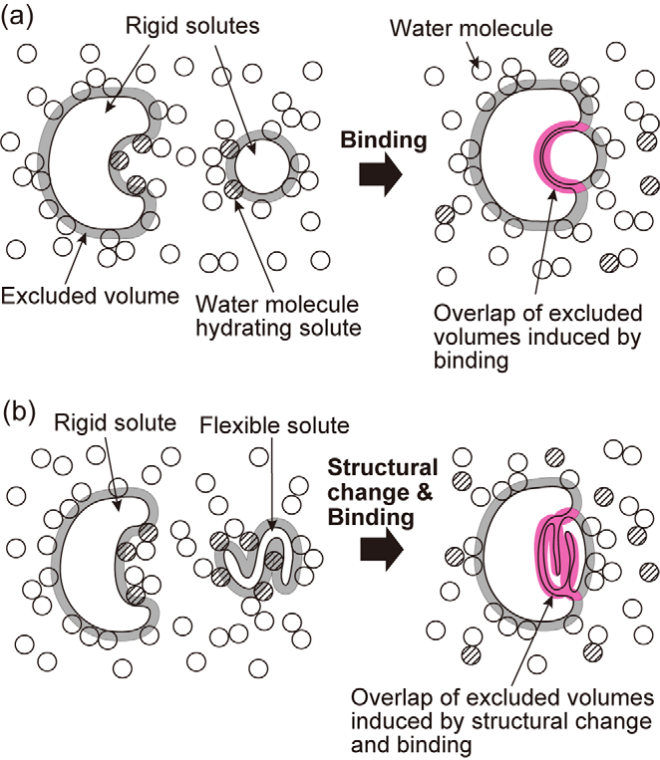
Cartoons illustrating the dehydration and the overlap of the excluded spaces accompanying the binding of two solute molecules. (**a**) Case where they are both rigid and their structures remain unchanged. (**b**) Case where one of them is flexible (i.e. structureless) and becomes structured after the binding.

The determination of the complex structure is an indispensable starting step toward the clarification of the driving force for the binding. However, it is not sufficient and to be followed by studies from the viewpoint of statistical thermodynamics of hydration. This is because the binding occurs in aqueous solution and water should play imperative roles. There are two principal points to be taken into account as illustrated in Figure 3. In the figure, the two solute molecules are rigid and their structures remain unchanged upon binding in (a), whereas one of them is structureless and becomes structured upon binding in (b). Firstly, the two molecules are hydrated, and the regions that become unexposed to water upon binding undergo dehydrations. When a solute region is hydrated, unless the region is predominantly nonpolar, the water structure near the region is significantly perturbed due to solute–water electrostatic and van der Waals attractive interactions. Upon dehydration, the attractive interactions are lost, giving rise to an energy increase. At the same time, the water structure is reorganized and the water–water electrostatic and van der Waals attractive interactions are recovered, leading to an energy decrease. The energy increase is approximately two times larger than the energy decrease (26,27) and the net change in energy is positive and considerably large, which is referred to as ‘energetic dehydration effect’ hereafter. Since a hydrogen bond is usually expressed as an electrostatic attractive interaction between hydrogen and oxygen or nitrogen, we consider that solute–solute, solute–water and water–water hydrogen bonds can be regarded as electrostatic attractive interactions. Secondly, each molecule generates an excluded space where the centers of water molecules cannot enter. The volume of the excluded space is referred to as the excluded volume (EV). Upon binding, the two excluded spaces overlap and the total EV decreases by the overlapped volume as shown in Figure 3a, which is followed by a corresponding increase in the total volume available to the translational displacement of water molecules in the system. Hence, the binding leads to a large gain of the configurational entropy of water. When the structure of one of the molecules changes to a more compact one upon binding as in the case of Figure 3b, the water-entropy gain becomes considerably larger than in the case of Figure 3a, because such a structural change results in a larger decrease in the total EV. We have shown that the physical factors described above, which can be categorized as ‘entropic EV effect’, play essential roles in a variety of biological self-assembly processes (28–32).

In the present study, we revisit the R12–P16 binding from the viewpoint of statistical thermodynamics of hydration. Since P16 does not take a particular tertiary structure when it is isolated, the binding is accompanied by a global structural change of P16. We remark that obtaining only microscopic information using a computer (e.g. molecular dynamics) simulation does not yield the clarification of the driving force. Calculating the changes of thermodynamic quantities upon binding is vitally important. We calculate the binding free energy using the molecular mechanics, hybrid method (30–37) in which the angle-dependent integral equation theory (ADIET) (38–42) applied to a multipolar water model (38,39) is combined with the morphometric approach (MA) (43,44) and 3D reference interaction site model (3D-RISM) theory (45–48). The ADIET and the 3D-RISM theory are statistical-mechanical theories for molecular liquids and hydration of solutes. The combination with the MA has recently been developed by us so that the ADIET can be applied to a complex biomolecule with drastic reduction in the computational load. The hybrid method and the 3D-RISM theory, which have been quite successful in analyzing a variety of self-assembly processes (27–32,49–52), are best suited to calculations of the hydration energy and entropy, respectively.

Our important findings are as follows. The binding is driven by a large gain of the water entropy; and the energy decrease due to the gain of R12–P16 attractive interactions is almost canceled out by the energy increase originating from the energetic dehydration effect upon binding. It should be noted, however, that the gain of R12–P16 attractive interactions is necessitated for compensating for the energetic dehydration effect. We point out that the general experimental observations manifesting precise stacking of flat moieties, electrostatic complementarity, including specific hydrogen bonding, and molecular shape complementarity can reasonably be interpreted. Further, we argue that the geometric characteristics (overall shapes, sizes and detailed polyatomic structures) of the solute molecules become the most important factors when the entropic EV effect dominates. This argument sheds new light on the elucidation of all types of molecular-recognition mechanisms within the same theoretical framework in a unified manner.

## MODEL AND THEORY

### Free-energy function

For a solute molecule immersed in water at infinite dilution, we define the free-energy function *G* expressed as

(1) \begin{equation*} G = E_{\rm C} - TS_{\rm C} + \mu _{\rm H} , \end{equation*}

where *E*_C_ and *S*_C_ are the conformational (intramolecular) energy and entropy of the solute molecule, respectively, *T* is the absolute temperature and *μ*_H_ is the hydration free energy defined as the excess chemical potential of the solute. We note that *μ*_H_ is independent of the solute insertion condition, isobaric or isochoric (53), and we consider isochoric condition that is much more convenient in a theoretical treatment. Using the relation,

(2) \begin{equation*} \mu _{\rm H} = \varepsilon _{{\rm VH}} - TS_{{\rm VH}} , \end{equation*}

where the subscript ‘VH’ denotes the hydration under isochoric condition, *ϵ*_VH_ and *S*_VH_ are the hydration energy and entropy, respectively, Equation (1) is rewritten as

(3) \begin{equation*} G = E_{\rm C} - TS_{\rm C} + \varepsilon _{{\rm VH}} - TS_{{\rm VH}}. \end{equation*}

Here, *ϵ*_VH_ represents the solute–water interaction energy generated plus the energy change due to the structural reorganization of water upon solute insertion and *S*_VH_ represents the change in the water entropy upon solute insertion. *G* is also independent of the solute insertion condition.

*E*_C_ is calculated on the basis of a molecular mechanical potential and thereby decomposed into the bonded, nonelectrostatic and electrostatic parts as

(4) \begin{equation*} E_{\rm C} = E_{\rm B} + E_{{\rm LJ}} + E_{{\rm ES}} , \end{equation*}

where *E*_B_ is the bond-type energy comprising the bond-stretching, angle-bending and torsion-angle energies, *E*_LJ_ is the Lennard–Jones (LJ) interaction energy and *E*_ES_ is the electrostatic interaction energy. We also decompose *ϵ*_VH_ as

(5) \begin{equation*} \varepsilon _{{\rm VH}} = \varepsilon _{{\rm VH},{\rm LJ}} + \varepsilon _{{\rm VH},{\rm ES}}, \end{equation*}

where *ϵ*_VH, LJ_ and *ϵ*_VH, ES_ are the nonelectrostatic and electrostatic contributions to *ϵ*_VH_, respectively. The decomposition is made in the following manner. First, we calculate the hydration energy of a hypothetical solute molecule whose partial charges are all switched to zero, *ϵ*_VH, LJ_. Then, we obtain *ϵ*_VH, ES_ from *ϵ*_VH, ES_ = *ϵ*_VH_ − *ϵ*_VH, LJ_.

Substituting Equations (4) and (5) into Equation (3) yields

(6a) \begin{equation*} G = E_{{\rm total}} - TS_{\rm C} - TS_{{\rm VH}} , \end{equation*}

(6b) \begin{equation*} E_{{\rm total}} = E_{\rm B} + (E_{{\rm LJ}} + \varepsilon _{{\rm VH},{\rm LJ}} ) + (E_{{\rm ES}} + \varepsilon _{{\rm VH},{\rm ES}} ). \end{equation*}

*E*_total_, which is referred to as the total energy hereafter, consists of *E*_B_ and the LJ and electrostatic components denoted by *E*_LJ_ + *ϵ*_VH, LJ_ and *E*_ES_ + *ϵ*_VH, ES_, respectively.

### Paths for calculating binding free energy

**Figure 4.**
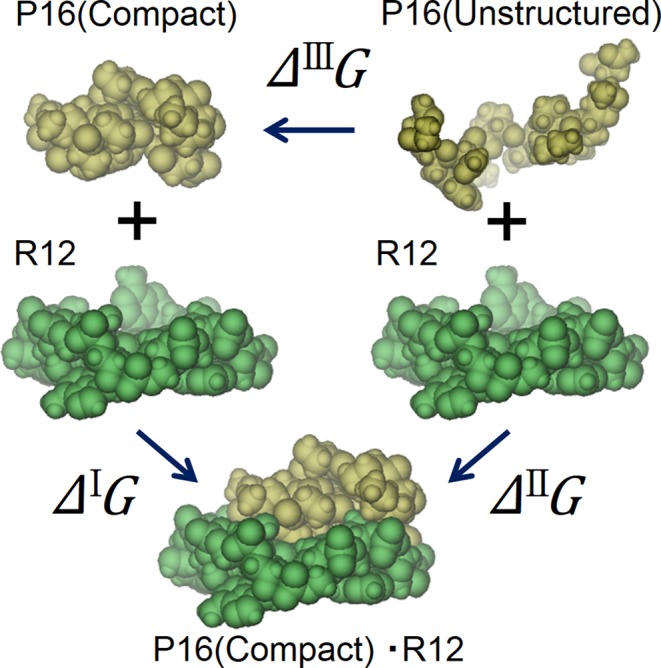
Schematic representation of the paths considered for calculating the binding free energy, the free-energy change upon the R12–P16 binding. The R12:P16 complex indicated by ‘P16(Compact)•R12’ is taken from an NMR structure (model 1) constructed as described in ‘Structure Modeling for R12:P16 Complex’. R12 and P16 are drawn on the upper and lower sides, respectively, using the space-filled model. *Δ*^I^*G*, *Δ*^II^*G* and *Δ*^III^*G* denote changes in the free-energy function along paths I, II and III, respectively. The water molecules are omitted here. This figure is drawn using the VMD.

The free-energy function and its constituents defined above are applicable to R12, P16 and the R12:P16 complex. Figure 4 shows a schematic representation of the paths considered for calculating the binding free energy, the free-energy change upon the R12–P16 binding. In path I, R12 and P16 in the complex are simply separated with no structural changes, and the resulting structures are employed as the isolated molecules. That is, it is assumed that both of R12 and P16 isolated possess unique structures with no structural fluctuations. In the real system, isolated P16 takes an ensemble of rather extended structures and undergoes a global structural change upon binding though R12 exhibits almost no change in its structure (5,23). This binding process is mimicked by path II. Path III represents the structural change of P16 from the unstructured state (P16_random coils_: it is assumed to be a set of random coils in the present study) to the compact structure taken from the complex (P16_compact_).

Let *Δ^M^X* (*M* = I, II, III) denote the change in *X* for path *M*. The binding free energies, which can be considered for paths I and II, are given by *Δ*^I^*G* and *Δ*^II^*G*, respectively. They are formally expressed as

(7) \begin{eqnarray*} &&\Delta ^{\rm I} G = G({\rm R}12:{\rm P}16) - \{ G({\rm R}12) + G({\rm P}16_{{\rm compact}} )\} \nonumber \\ &&= \Delta ^{\rm I} (E_{{\rm LJ}} + \varepsilon _{{\rm VH},{\rm LJ}} ) + \Delta ^{\rm I} (E_{{\rm ES}} + \varepsilon _{{\rm VH},{\rm ES}} ) - T\Delta ^{\rm I} S_{{\rm VH}} , \end{eqnarray*}

(8) \begin{eqnarray*} &&\Delta ^{{\rm II}} G = G({\rm R}12:{\rm P}16) - \{ G({\rm R}12) + G({\rm P}16_{{\rm random}\;{\rm coils}} )\} \nonumber \\ &&= \Delta ^{{\rm II}} E_{\rm B} + \Delta ^{{\rm II}} (E_{{\rm LJ}} + \varepsilon _{{\rm VH},{\rm LJ}} ) + \Delta ^{{\rm II}} (E_{{\rm ES}} + \varepsilon _{{\rm VH},{\rm ES}} ) \nonumber \\ &&- T\Delta ^{{\rm II}} S_{\rm C} - T\Delta ^{{\rm II}} S_{{\rm VH}}. \end{eqnarray*}

*G*(R12), for example, is the free-energy function for R12. *Δ*^I^*E*_ES_, *Δ*^I^*ϵ*_VH, LJ_ and *Δ*^I^*ϵ*_VH, ES_, for example, originate from electrostatic attractive interactions between R12 and P16, from the energetic dehydration effect due to the loss of R12–water and P16–water van der Waals attractive interactions and from the energetic dehydration effect due to the loss of R12–water and P16–water electrostatic attractive interactions, respectively, and *Δ*^I^*S*_VH_ represents the water-entropy gain in the binding process of path I. We remark that about half of the energy increase arising from the loss of R12–water and P16–water attractive interactions is canceled out by the energy decrease due to the structural reorganization of water upon binding (see the fourth paragraph of the Introduction section). In path I, *Δ*^I^*E*_B_ = 0 and *Δ*^I^*S*_C_ = 0. The free-energy change due to the global structural change of P16, *Δ*^III^*G*, is given by

(9) \begin{eqnarray*} &&\Delta ^{{\rm III}} G = G({\rm P}16_{{\rm compact}} ) - G({\rm P}16_{{\rm random}\,{\rm coils}} ). \nonumber \\ &&= \Delta ^{{\rm III}} E_{\rm B} + \Delta ^{{\rm III}} (E_{{\rm LJ}} + \varepsilon _{{\rm VH},{\rm LJ}} ) + \Delta ^{{\rm III}} (E_{{\rm ES}} + \varepsilon _{{\rm VH},{\rm ES}} ) \nonumber \\ &&- T\Delta ^{{\rm III}} S_C - T\Delta ^{{\rm III}} S_{{\rm VH}}. \end{eqnarray*}

It should be noted that *Δ*^II^*G* = *Δ*^I^*G* + *Δ*^III^*G* and *Δ*^II^*S*_C_ = *Δ*^III^*S*_C_.

Since P16_random coils_ is more stable than P16_compact_, *Δ*^III^*G* > 0. *Δ*^II^*G* < 0 and it follows that *Δ*^I^*G* < 0 and │*Δ*^I^*G*│ > │*Δ*^II^*G*│. *Δ*^II^*S*_C_ < 0 and −*TΔ*^II^*S*_C_ ( = −*TΔ*^III^*S*_C_) cannot be a driving force for the binding. *Δ*^II^*E*_B_ ( = *Δ*^III^*E*_B_) should have a relatively small contribution to the binding free energy. We present the theoretical results for *Δ*^II^*G* and *Δ*^III^*G* by omitting −*TΔ*^II^*S*_C_ for the following reasons: the calculation of conformational entropy is a formidable task and modeling the unstructured state of isolated P16 is accompanied by significant uncertainty. However, the validity of our theoretical method can be checked in the following manner. The dissociation constant for the R12–P16 binding was determined by Mashima *et al.* (5) through a filter binding assay experiment by assuming that P16 bound to an R12 monomer. However, R12 is present as a dimer and two P16s bind to the R12 molecules forming the dimer, respectively (see Figure 1). In the present study, we recalculate the dissociation constant (the recalculated one is denoted by *k*_D_) as described in Section A of Supplementary Appendices and obtain an approximate value of the binding free energy corresponding to *Δ*^II^*G* as −*RT*ln(*k*_D_) that is denoted by *Δ*^II^*G*_experimental_ (*R* is the gas constant). The theoretical value of *Δ*^II^*G* in which −*TΔ*^II^*S*_C_ is omitted, *Δ*^II^*G*_theoretical_, must be sufficiently lower than *Δ*^II^*G*_experimental_. This criterion is certainly satisfied as shown in the Results and Discussion section (−*TΔ*^II^*S*_C_ is roughly estimated and a discussion is also made on the quantitative aspect of the theoretical results).

Our principal concern is to examine the signs and magnitudes of *Δ^M^*(*E*_LJ_ + *ϵ*_VH, LJ_), *Δ^M^*(*E*_ES_ + *ϵ*_VH, ES_) and −*TΔ^M^S*_VH_ (*M* = I, II) which enable us to specify the driving force. Those for path I may be better suited to quantitative analyses because the binding free energy for path I given by Equation (7) does not include changes in the bond-type energy and the solute conformational entropy.

### Calculation of hydration entropy

The ADIET (38–42) applied to a multipolar model for water (38,39) has been quite successful in studies on hydrophobic and hydrophilic hydrations. In the theory, the orientational dependency of the water–water and solute–water interaction potentials and correlations is explicitly taken into consideration. In most of our studies, a water molecule is modeled as a hard sphere with diameter *d*_S_ = 0.28 nm in which a point dipole and a point quadrupole of tetrahedral symmetry are embedded. The effect of the molecular polarizability is taken into account using the self-consistent mean field (SCMF) theory (38,39). At the SCMF level, the many-body-induced interactions are reduced to pairwise additive potentials involving an effective dipole moment. The effective dipole moment thus determined is about 1.42 times larger than the bare gas-phase dipole moment. The dielectric constant of bulk water, which is a good measure of the validity of a theory, is calculated to be ∼83 that is in good agreement with the experimental value ∼78. Further, the theory is capable of reproducing the solubility of methane featuring the minimum at about 350 K (41). The hydration free energy of a spherical, nonpolar solute calculated is in perfect agreement with that obtained by computer simulations using more popular water models such as the transferable intermolecular potential four point (TIP4P) and the extended single point charge (SPC/E) (41). A more detailed description of the ADIET is given in Section B of Supplementary Appendices.

It is not straightforward to apply the ADIET to a large, complex solute with polyatomic structure due to the mathematical complexity. Fortunately, as far as the hydration entropy *S*_VH_ is concerned, it has been shown to be rather insensitive to the solute–water interaction potentials. For example, Imai *et al.* (47) examined *S*_VH_ for a total of eight peptides and proteins using the 3D-RISM theory combined with the all-atom potentials and found the following: even when the protein–water electrostatic interactions, which are quite strong, are completely shut off and only the LJ interactions are retained, *S*_VH_ changes merely by less than 5%. Thus, only the geometric characteristics are essential in the calculation of *S*_VH_, and a solute molecule can be modeled as a set of fused, neutral hard spheres. The diameter of an atom in a solute molecule is set at the corresponding *σ*-value for the LJ potential. As a consequence, the MA (43,44) becomes a very powerful tool. We employ the hybrid method in which the ADIET is combined with the MA for calculating *S*_VH_.

The idea of the MA is to express *S*_VH_ by the linear combination of only four geometric measures of a solute molecule:

(10) \begin{equation*} S_{{\rm VH}} /k_{\rm B} = C_1 V_{{\rm ex}} + C_2 A + C_3 X + C_4 Y. \end{equation*}

Here, Equation (10) is referred to as the morphometric form, *V*_ex_ is the EV generated by the solute molecule, *A* is the water-accessible surface area, *X* and *Y* are the integrated mean and Gaussian curvatures of the water-accessible surface, respectively, and *k*_B_ is the Boltzmann constant. The water-accessible surface is the surface that is accessible to the centers of water molecules. The EV, as defined in the previous section, is the volume that is enclosed by this surface. We note that *C*_1_ is completely independent of the solute–water interaction potential. Though *S*_VH_ is influenced by all the four terms, *C*_1_*V*_ex_ is the principal term at normal temperature and pressure. This is the reason for the fair insensitivity of *S*_VH_ to the solute–water interaction potential. The contribution from the water molecules near the solute molecule is represented by the other three terms. In the MA, the solute shape enters *S*_VH_ only via the four geometric measures. Therefore, the four coefficients (*C*_1_–*C*_4_) can be determined in simple geometries: they are calculated from the values of *S*_VH_ of hard-sphere solutes immersed in our model water. The ADIET is employed in the calculation. The four coefficients are determined by the least square fitting applied to the following equation (i.e. Equation 10 applied to hard-sphere solutes):

(11) \begin{eqnarray*} \begin{array}{*{20}l} {S_{{\rm VH}} /k_{\rm B} = } \\ {C_1 (4\pi R^3 /3) + C_2 (4\pi R^2 ) + C_3 (4\pi R) + C_4 (4\pi ),} \\ {R = (d_{\rm U} + d_{\rm S} )/2.} \\ \end{array} \end{eqnarray*}

Here, *d*_U_ denotes the hard-sphere diameter and sufficiently many different values of *d*_U_ are considered. *T* is set at 298 K, and the number density of bulk water *ρ*_S_ is taken to be that of real water on the saturation curve, *ρ*_S_ = 0.0333 Å^−3^. Once the four coefficients are determined, *S*_VH_ of the solute molecule with a prescribed structure is obtained from Equation (10) only if the four geometric measures are calculated.

The high reliability of the hybrid method in calculating *S*_VH_ has been demonstrated in the following examples wherein the water-entropy effect is treated as the key factor: quantitative reproduction of the experimentally measured changes in thermodynamic quantities upon apoplastocyanin (apoPC) folding (30); elucidation of the microscopic mechanisms of pressure (33) and cold (34,35) denaturating of a protein; and proposal of a reliable measure of the thermodynamic stability of a protein (36,37).

### Calculation of hydration energy

The hydration energy *ϵ*_VH_, which is largely dependent on the solute–water interaction potential, cannot be calculated by the hybrid method described above. We employ the 3D-RISM theory (45–48) for the calculation of *ϵ*_VH_. The LJ potential parameters and partial charges are assigned to the atoms constituting the solute molecules. The 3D-RISM theory has successfully been applied to important problems in biological systems such as the hydration properties of peptides and proteins (27,47), receptor-ligand binding processes (49,50) and association of protein molecules (51,52). However, the theory is not very good at elucidating the hydration of a hydrophobic molecule. For instance, the theory underestimates the absolute value of the water-entropy change upon protein folding though the hydration-energy change remains quantitatively reliable (27). Taken together, the 3D-RISM theory and the hybrid method are best suited to calculations of *ϵ*_VH_ and *S*_VH_, respectively.

A description of the 3D-RISM theory is provided in Section C of Supplementary Appendices. First, the site–site correlation functions for bulk water are calculated using the dielectrically consistent RISM (DRISM) theory (54,55) coupled with the Kovalenko–Hirata (KH) closure equation (46). The site–site intermolecular potentials, water density (*ρ*_S_ = 0.0333 Å^−3^) and absolute temperature (*T* = 298 K) are served as the input data. The SPC/E model (56) is employed for water with a correction in terms of the LJ potential parameters for the hydrogen sites (*σ* = 0.654 Å, *ϵ* = 0.0155 kcal/mol). The water–solute correlation functions are then obtained for a solute molecule with a prescribed structure by solving the 3D-RISM/KH equations. The site–site correlation functions for bulk water and the water–solute interaction potentials form the input data. The LJ potential parameters and partial charges for the solute atoms are taken from the standard Amber99SB force field (57). The solution is performed on a 3D cubic grid. The grid spacing (*Δx, Δy* and *Δz*) is set at 0.5 Å, and the grid resolution (*N_x_*×*N_y_*×*N_z_*) is 256 × 256 × 256. It has been confirmed that the spacing is sufficiently small and the box size (*N_x_Δx*, *N_y_Δy*, *N_z_Δ*z) is large enough for the result obtained to be identical within convergence tolerance. The hydration free energy *μ*_H_ is calculated using the 3D extension of the Singer–Chandler formula adapted to the KH closure equation (46,58). Finally, *ϵ*_VH_ is obtained from *ϵ*_VH_ = *μ*_H_ + *TS*_VH_, where *S*_VH_ is numerically evaluated as the temperature derivative of *μ*_H_.

### Structure modeling of R12:P16 complex

The ten 3D structures of the 2×R12:2×P16 complex were taken from the Protein Data Bank (PDB code: 2RSK). They were determined by Mashima *et al.* (5) using a simulated annealing protocol combined with the interatomic distance and dihedral angle constraints obtained from the nuclear magnetic resonance (NMR) experiments. In the simulated annealing protocol, the *σ*-values in the LJ potentials of the Amber99SB force field (57) were multiplied by 0.7 to widely explore the structural space for the complex.

We employ an advanced molecular mechanics refinement procedure using the Amber99SB force field with the full *σ*-values. This procedure, which utilizes the generalized Born and surface area (GB/SA) model solvent (59,60), is combined with the interatomic distance constraints obtained from the nuclear Overhauser effect spectroscopy (NOESY). NOESY intensities are taken into account as the limits of interatomic distances. These limits are about 1.4 ( = 1/0.7) times larger than those employed by Mashima *et al.* in the previous study (5). The ten structures are refined with 100 steps of the steepest-descent energy minimization followed by the conjugated gradient minimization which is continued until the root mean square forces acting on the complex atoms become weaker than 1.0 × 10^−4^ (kcal/mol)/Å. All the calculations are carried out using the AMBER12 molecular dynamics package (61).

Although the effect of water is further taken into account in the present refinement procedure, the important structural features of the 3D structures determined by Mashima *et al.* (5) are conserved quite well. Example common features are the stacking between a guanine nucleotide in R12 and an indole ring of tryptophan in P16 and the contact of phosphate groups in R12 with lysine residues in P16 (see Figure 2).

Since the upper and lower halves of the 2×R12:2×P16 complex share almost the same 3D structure, the upper half is taken from each complex structure thus constructed and used as the structure of the R12:P16 complex. We calculate the free-energy function where the conformational entropy is set at zero for each model structure of the R12:P16 complex. The ten models obtained are ranked in ascending order with respect to this free-energy function, and the first to fifth models (models 1 through 5) are chosen for the present study. The coordinates of the ensemble of five structures thus obtained for the 2×R12:2×P16 complex have been deposited in the Protein Data Bank with the accession code 2RU7.

### Structure modeling of P16 in unstructured state

The unstructured state of P16 is modeled as a set of random coils. First, P16 is taken from the R12:P16 complex and its structure is refined with 100 steps of the steepest-descent energy minimization followed by the conjugated gradient minimization with the GB/SA model solvent which is continued until the root mean square forces acting on the P16 atoms become weaker than 1.0 × 10^−4^ (kcal/mol)/Å. Then, starting from the structure thus obtained, we assign random numbers to the dihedral angles for the backbone chain, *Φ* and *Ψ*. The random numbers are limited to the range from −180° to −30° for *Φ* and from −180° to −150° and from −90° to 180° for *Ψ* which correspond to the allowed regions in the Ramachandran map. For glycine, *Φ* is distributed from −180° to −30° and from 30° to 180°, and *Ψ* is from −180° to 180°. For proline, *Φ* is set at −65° and *Ψ* is set at 180°. The 200 models, which are obtained in this way, are refined in accordance with the minimization techniques described above. The models that give divergently high LJ potential energies due to the overlap of the constituent atoms are then excluded, and the remaining 103 models are employed as the random coils. All calculations are carried out using the AMBER12 molecular dynamics package (61). The average value of the free-energy function for these models is regarded as *G*(P16_random coils_).

## RESULTS AND DISCUSSION

### Gain of water entropy upon binding: driving force

Table 1 gives the binding free energy and its two components, total-energy change and contribution from the water-entropy gain, upon the R12–P16 binding in path I (see Figure 4). The binding free energy, which is in the range from −47.05 to −30.31 kcal/mol, is governed by the contribution from the water-entropy gain for all the five models of the complex structure. The total-energy change is positive and against the binding for models 1, 3 and 4. It is negative and in favor of the binding for models 2 and 5, but the absolute value of the contribution from the water-entropy gain is roughly an order of magnitude larger than that of the total-energy change. We can conclude that the binding is driven by the water-entropy gain.

**Table 1.** Binding free energy and its energetic and entropic components (in kcal/mol) upon the R12–P16 binding in path I

Model	*Δ*^I^*G*	*Δ*^I^*E*_total_	−*TΔ*^I^*S*_VH_
1	−36.16	5.47	−41.63
2	−39.80	−3.10	−36.71
3	−35.84	0.14	−35.98
4	−30.31	3.43	−33.73
5	−47.05	−6.58	−40.47

Five different models, which correspond to the upper halves of the 2×R12:2×P16 complex structures in the Protein Data Bank with the accession code 2RU7, are considered for the structure of the R12:P16 complex.

Binding free energy, *Δ*^I^*G* = *G*(R12:P16) − {*G*(R12) + *G*(P16_compact_)} = *Δ*^I^*E*_total_ − *TΔ*^I^*S*_VH_.

Total-energy change, *Δ*^I^*E*_total_ = *Δ*^I^(*E*_LJ_ + *ϵ*_VH, LJ_) + *Δ*^I^(*E*_ES_ + *ϵ*_VH, ES_).

Contribution from the water-entropy gain, −*TΔ*^I^*S*_VH_.

Table 2 gives the binding free energy and its two components upon the R12–P16 binding in path II. Unlike in path I, the structural change of P16 accompanying the binding is taken into account in path II. The binding free energy is in the range from −23.75 to −4.59 kcal/mol. The total-energy change takes a considerably large, positive value for all the five models of the complex structure, and it strongly opposes the binding. However, the water-entropy gain surpasses the increase in the total energy and drives the binding. Physicochemical interpretations of the results displayed in the two tables are provided in later sections.

**Table 2.** Binding free energy and its energetic and entropic components (in kcal/mol) upon the R12–P16 binding in path II

Model	*Δ*^II^*G*	*Δ*^II^*E*_total_	−*TΔ*^II^*S*_VH_
1	−21.67	36.26	−57.93
2	−14.04	40.32	−54.36
3	−23.75	27.43	−51.18
4	−4.59	45.10	−49.70
5	−11.53	48.84	−60.37

Five different models, which correspond to the upper halves of the 2×R12:2×P16 complex structures in the Protein Data Bank with the accession code 2RU7, are considered for the structure of the R12:P16 complex.

Binding free energy, *Δ*^II^*G* = *G*(R12:P16) − {*G*(R12) + *G*(P16_random coils_)}, where −*TΔ*^II^*S*_C_ is omitted: *Δ*^II^*G* = *Δ*^II^*E*_total_ − *TΔ*^II^*S*_VH_.

Total-energy change, *Δ*^II^*E*_total_ = *Δ*^II^*E*_B_ + *Δ*^II^(*E*_LJ_ + *ϵ*_VH, LJ_) + *Δ*^II^(*E*_ES_ + *ϵ*_VH, ES_).

Contribution from the water-entropy gain, −*TΔ*^II^*S*_VH_.

### Comparison between theoretical and experimental values of binding free energy

We compare the binding free energy calculated for path II, *Δ*^II^*G*_theoretical_ (*Δ*^II^*G* in Table 2), with the experimentally determined value, *Δ*^II^*G*_experimental_. *Δ*^II^*G*_experimental_ can be obtained as −*RT*ln(*k*_D_) where *k*_D_ is the dissociation constant for the R12–P16 binding: *Δ*^II^*G*_experimental_ = −6.45 kcal/mol (see Section A of Supplementary Appendices). In a strict sense, *Δ*^II^*G*_theoretical_ cannot be in complete agreement with *Δ*^II^*G*_experimental_. This is because for *Δ*^II^*G*_experimental_ = −*RT*ln(*k*_D_) the standard state is 1 mol/l and the activity coefficient is set at unity while in the theoretical calculation the standard state is the infinite dilution (62). Nevertheless, the two values should be close to each other. As stated above, *Δ*^II^*G*_theoretical_ in which −*TΔ*^II^*S*_C_ is omitted must be sufficiently lower than *Δ*^II^*G*_experimental_. This requirement is certainly met except in the case of model 4.

Here, it is worthwhile to roughly estimate −*TΔ*^II^*S*_C_. Since R12 undergoes essentially no structural change upon the R12–P16 binding, *Δ*^II^*S*_C_ is almost equal to the conformational-entropy loss for P16 caused by the transition from the random-coil state to the compact structure, *Δ*^III^*S*_C_. Fitter (63) studied the conformational-entropy loss upon protein folding using neutron spectroscopy. He estimated the temperature dependence of the radius parameter *r* representing a length scale within which each residue can freely move. The loss is given by −3*k*_B_*N*_r_ln(*r*_u_/*r*_f_) where *r*_u_ and *r*_f_ are the radius parameters for the unfolded and folded states, respectively, and *N*_r_ is the number of residues. The estimation of the radius parameters was made at three temperatures: 303, 323 and 343 K. In order to obtain the loss at 298 K, we first performed the linear fitting of the temperature dependence of *r*_u_ and *r*_f_, and then calculated their values at 298 K. The loss for P16 with *N*_r_ = 12 was thus obtained: −*TΔ*^II^*S*_C_ = −*TΔ*^III^*S*_C_ ∼ 8.83 kcal/mol. The neutron scattering experiments cover only the picosecond time regime although the fluctuations in other time scales also affect the loss (63). For this reason, our method based on Fitter's experiments tends to give significant underestimation of the loss. However, P16 possesses two proline residues, making its random coils rather compact, with the result of an exceptionally small loss. For this reason, it may be justified to adopt the above result as a rough estimation.

If the contribution from the conformational-entropy loss, −*TΔ*^II^*S*_C_ = 8.83 kcal/mol, is incorporated in the theoretical calculation for the binding free energy, the result is in the range from −14.92 to 4.24 kcal/mol. The average value is −6.29 kcal/mol which is comparable with *Δ*^II^*G*_experimental_ = −6.45 kcal/mol. We can conclude that our theoretical results are reliable even in a quantitative sense. (Model 4 might be inappropriate because it gives the binding free energy a positive value when –*TΔ*^II^*S*_C_ is incorporated.)

### Decomposition of total-energy change into various components

The total-energy change upon the R12–P16 binding in path I is decomposed into *Δ*^I^*E*_LJ_, *Δ*^I^*ϵ*_VH, LJ_, *Δ*^I^*E*_ES_ and *Δ*^I^*ϵ*_VH, ES_ as displayed in Table 3. *Δ*^I^*E*_LJ_ and *Δ*^I^*ϵ*_VH, LJ_ are both significantly large, but the former is negative while the latter is positive. *Δ*^I^*E*_ES_ and *Δ*^I^*ϵ*_VH, ES_ are even much larger, but the former is negative while the latter is positive. *Δ*^I^*E*_LJ_ and *Δ*^I^*ϵ*_VH, LJ_ or *Δ*^I^*E*_ES_ and *Δ*^I^*ϵ*_VH, ES_ are compensating. Thus, the binding gives rise to an energy decrease arising from van der Waals attractive interactions between R12 and P16, but it is accompanied by an energy increase related to the loss of R12–water and P16–water van der Waals attractive interactions. The former is slightly larger and *Δ*^I^(*E*_LJ_ + *ϵ*_VH, LJ_) takes a rather small, negative value. The binding brings an energy decrease arising from electrostatic attractive interactions between R12 and P16, but it is accompanied by an energy increase related to the loss of R12–water and P16–water electrostatic attractive interactions. The latter is slightly larger and *Δ*^I^(*E*_ES_ + *ϵ*_VH, ES_) takes a rather small, positive value. As a matter of fact, the loss of R12–water and P16–water attractive interactions is quite large. However, about half of it is canceled out by the water reorganization energy (26,27) (i.e. the energy lowering due to the structural reorganization of water upon binding) with the result that the total loss becomes comparable with the gain of R12–P16 attractive interactions. *Δ*^I^(*E*_LJ_ + *ϵ*_VH, LJ_) and *Δ*^I^(*E*_ES_ + *ϵ*_VH, ES_) are also compensating, leading to a considerably small total-energy change (it is either positive or negative).

**Table 3.** Components of total-energy change (in kcal/mol) upon the R12–P16 binding in path I

Model	*Δ*^I^*E*_total_	*Δ*^I^(*E*_LJ_ + *ϵ*_VH, LJ_)	*Δ*^I^(*E*_ES_ + *ϵ*_VH, ES_)	*Δ*^I^*E*_LJ_	*Δ*^I^*ϵ*_VH, LJ_	*Δ*^I^*E*_ES_	*Δ*^I^*ϵ*_VH, ES_
1	5.47	−3.94	9.41	−54.92	50.98	−1048.45	1057.85
2	−3.10	−6.33	3.23	−52.84	46.51	−1017.00	1020.23
3	0.14	−2.05	2.19	−50.55	48.50	−1070.42	1072.61
4	3.43	−5.04	8.47	−50.81	45.77	−1001.51	1009.98
5	−6.58	−9.55	2.97	−53.50	43.95	−1021.89	1024.85

Five different models, which correspond to the upper halves of the 2×R12:2×P16 complex structures in the Protein Data Bank with the accession code 2RU7, are considered for the structure of the R12:P16 complex.

Total-energy change, *Δ*^I^*E*_total_ = *Δ*^I^(*E*_LJ_ + *ϵ*_VH, LJ_) + *Δ*^I^(*E*_ES_ + *ϵ*_VH, ES_), comprises *Δ*^I^*E*_LJ_, *Δ*^I^*ϵ*_VH, LJ_, *Δ*^I^*E*_ES_ and *Δ*^I^*ϵ*_VH, ES_.

*Δ*^I^(*E*_LJ_ + *ϵ*_VH, LJ_) is the LJ component.

*Δ*^I^(*E*_ES_ + *ϵ*_VH, ES_) is the electrostatic component.

*Δ*^I^*E*_LJ_,

*Δ*^I^*ϵ*_VH,__LJ_,

*Δ*^I^*E*_ES_, and

*Δ*^I^*ϵ*_VH, ES_ originate from van der Waals attractive interactions between R12 and P16, from electrostatic attractive interactions between R12 and P16, from the energetic dehydration effect related to the loss of R12–water and P16–water van der Waals attractive interactions and from the energetic dehydration effect related to the loss of R12–water and P16–water electrostatic attractive interactions, respectively. The contribution from the structural reorganization of water upon binding is included in *Δ*^I^*ϵ*_VH, LJ_ and *Δ*^I^*ϵ*_VH, ES_.

The energy decrease due to the gain of R12–P16 attractive interactions is almost canceled out by the energy increase caused by the energetic dehydration effect. The binding is driven by the entropic EV effect with the help of the gain of R12–P16 attractive interactions compensating the dehydrations. A loss of the conformational entropy of P16 opposes the binding, but this effect is much smaller than the driving force, i.e. the water-entropy gain.

### Global structural change of P16 upon binding

Table 4 gives the free-energy change, total-energy change and contribution from the water-entropy gain arising from the global structural change of P16 referred to as path III (see Figure 4). The reason for the significantly large total-energy change can be summarized as follows. The energy decrease, which arises from intramolecular van der Waals attractive interactions brought by the structural change, is somewhat compensated by the energy increase related to the loss of P16–water van der Waals attractive interactions. As a consequence, *Δ*^III^(*E*_LJ_ + *ϵ*_VH, LJ_) takes a rather small, negative value. By contrast, the energy increase related to the loss of P16–water electrostatic attractive interactions is considerably larger than the energy decrease arising from intramolecular electrostatic attractive interactions brought by the structural change. As a consequence, *Δ*^III^(*E*_ES_ + *ϵ*_VH, ES_) takes a considerably large, positive value, giving rise to the significantly large total-energy change. The water-entropy gain contributes to the free-energy change as a negative component, but this effect is smaller than the positive total-energy change. Thus, the free-energy change takes a significantly large, positive value. It increases further when the conformational-entropy loss, −*TΔ*^III^*S*_C_ = 8.83 kcal/mol, is incorporated. The positive free-energy change in path III is consistent with the experimental observation that isolated P16 is characterized by no well-defined structure.

**Table 4.** Free-energy change and its energetic and entropic components (in kcal/mol) due to the global structural change of P16 upon the R12–P16 binding referred to as path III

Model	*Δ*^III^*G*	*Δ*^III^*E*_total_	−*TΔ*^III^*S*_VH_
1	14.49	30.79	−16.30
2	25.76	43.42	−17.65
3	12.09	27.29	−15.19
4	25.71	41.68	−15.96
5	35.52	55.42	−19.90

Five different models are considered for the structure of P16_compact_ (P16_compact_ is taken from the R12:P16 complex).

Free-energy change due to the global structural change of P16 upon the R12–P16 binding, *Δ*^III^*G* = *G*(P16_compact_) − *G*(P16_random coils_), where −*TΔ*^III^*S*_C_ ( = −*TΔ*^II^*S*_C_) is omitted: *Δ*^III^*G* = *Δ*^III^*E*_total_ −*TΔ*^III^*S*_VH_.

Total-energy change, *Δ*^III^*E*_total_ = *Δ*^III^*E*_B_ + *Δ*^III^(*E*_LJ_ + *ϵ*_VH, LJ_) + *Δ*^III^(*E*_ES_ + *ϵ*_VH, ES_).

Contribution from the water-entropy gain, −*TΔ*^III^*S*_VH_.

The large electrostatic dehydration effect can be interpreted as follows. The amino-acid sequence of P16 is Gly-Gln-Trp-Asn-Lys-Pro-Ser-Lys-Pro-Lys-Thr-Asn. Except for Gly, P16 possesses 11 side chains. Three of them (three lysine residues) are positively charged, six of them (Gln, Trp, Asn, Ser, Thr and Asn) are polar and only two of them (two proline residues) are nonpolar. P16 is thus highly hydrophilic and substantially more stabilized when the water-accessible surface area is large, i.e. it is rather extended.

### Physical origins of water-entropy gains upon R12–P16 binding

The principal term in the right hand of Equation (10) is the first one dependent on the EV at normal temperature and pressure. The water-entropy gain upon the R12–P16 binding can be discussed by decomposing it into the water-entropy gains accompanying paths III and I in Figure 4: (III) the global structural change of P16 from a random-coil state to the compact structure and (I) the contact of P16 possessing the compact structure with R12. In path III, many of the atoms in P16 contact one another, leading to a large decrease in the EV. In path I, the atoms of P16 and R12 at their interface are closely packed, leading to an even larger decrease in the EV. The EV decreases by 12.49*d*_S_^3^ (*d*_S_ is the molecular diameter of water, 0.28 nm) in path III and by 33.33*d*_S_^3^ in path I. These are the average values for the five different models of the R12:P16 complex. Since *Δ*^III^*V*_ex_/*d*_S_^3^ = −12.49, *Δ*^I^*V*_ex_/*d*_S_^3^ = −33.33 and *C*_1_<0, the R12–P16 binding accompanied by the structural change of P16 leads to a large gain of the water entropy.

### Changes in hydration entropy and enthalpy under isobaric condition

The theoretical calculations are made under isochoric condition while the experiments are performed under isobaric condition. The changes in hydration quantities under the two conditions are related to one another as (53,64)

(12a) \begin{equation*} \Delta H/(k_{\rm B} T) = \Delta \varepsilon _{{\rm VH}} /(k_{\rm B} T) + (\alpha ^*; /\kappa _T^* )\Delta V_{{\rm PH}} /d_{\rm S}^3 , \end{equation*}

(12b) \begin{equation*} \Delta S_{{\rm PH}} /k_{\rm B} = \Delta S_{{\rm VH}} /k_{\rm B} + (\alpha ^* /\kappa _T^* )\Delta V_{{\rm PH}} /d_{\rm S}^3 , \end{equation*}

(13) \begin{eqnarray*} &&\Delta \mu _{\rm H} /(k_{\rm B} T) = \Delta \varepsilon _{{\rm VH}} /(k_{\rm B} T) - \Delta S_{{\rm VH}} /k_{\rm B} = \nonumber \\ &&\Delta H/(k_{\rm B} T) - \Delta S_{{\rm PH}} /k_{\rm B}. \end{eqnarray*}

Here, the subscript ‘PH’ denotes the hydration under isobaric condition, *H* is the hydration enthalpy, *ΔV*_PH_ is the system-volume change and *d*_S_ is the molecular diameter of water. α* and κ_T_*, which depend only on the properties of pure water, are defined as

(14a) \begin{equation*} \alpha ^* = \alpha T, \end{equation*}

(14b) \begin{equation*} \kappa _T^* = \kappa _T k_{\rm B} T/d_{\rm S}^3 , \end{equation*}

where *α* is the isobaric thermal expansion coefficient and *κ_T_* is the isothermal compressibility. *ΔV*_PH_ can be calculated by the 3D-RISM theory (45–48). The values obtained are *ΔV*_PH_/*d*_S_^3^ = 5.51, 5.47 and −0.04 in paths I, II and III, respectively. *k*_B_*T*(α*/κ_T_*)*ΔV*_PH_/*d*_S_^3^ in path I, for example, is only 2.92 kcal/mol that is far smaller than *Δ*^I^*S*_VH_, *Δ*^I^*ϵ*_VH, LJ_ and *Δ*^I^*ϵ*_VH, ES_. Thus, the changes in hydration quantities under isobaric condition are almost indistinguishable from those under isochoric condition.

It is worthwhile to comment on a general case. Upon binding of a hydrophobic receptor and a hydrophobic ligand under isobaric condition, for example, the compression of bulk water occurs, possibly leading to a water-entropy loss (31): due to considerably large, negative *ΔV*_PH_, *ΔS*_PH_ can become negative despite positive *ΔS*_VH_. Of course, the water entropy always increases upon the binding under isochoric condition. Thus, isochoric condition, which is free from the effects of compression or expansion of bulk water, is more suited to the physical interpretation of a change in a thermodynamic quantity of hydration.

### Significance of electrostatic complementarity

In the R12:P16 complex, the negatively charged phosphate groups of R12 are in contact with the positively charged ϵ-amino groups of lysine residues of P16, leading to R12–P16 energetic stabilization brought by electrostatic attractive interactions (see Figure 2) (5). This is a typical example of the electrostatic complementarity (2). Before the R12–P16 binding, the negatively and positively charged groups interact with water hydrogens with positive partial charges and with water oxygens with negative partial charges, respectively, maintaining R12–water and P16–water electrostatic attractive interactions. The binding is driven by the water-entropy gain, but it is unavoidably accompanied by an energy increase arising from the loss of R12–water and P16–water electrostatic attractive interactions. About half of the energy increase is canceled out by the energy decrease due to the structural reorganization of water upon binding (26,27), but the net effect acts as destabilization. This destabilization and the R12–P16 energetic stabilization are compensating. In other words, the latter is required for canceling the former. Similar arguments can be made for general binding processes for two solute molecules, and this is why the electrostatic complementarity is often observed in a complex. Looking at the structural data for a complex without accounting for the water roles can mislead to the conclusion that the R12–P16 binding, for instance, is driven by the R12–P16 electrostatic attractive interactions described above.

**Figure 5.**
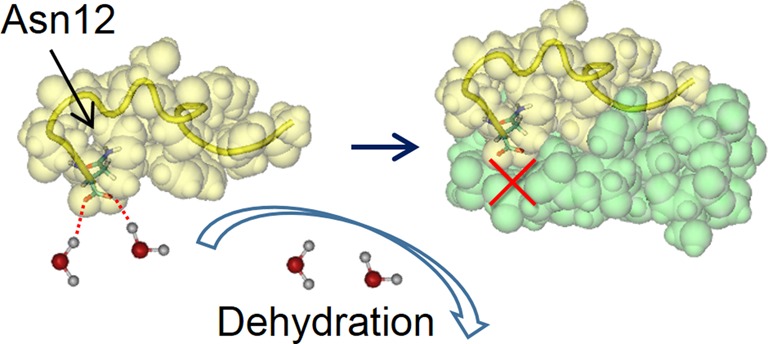
Image illustrating an example imperfection of the electrostatic complementarity in the R12:P16 complex. R12 and P16 are drawn on the upper and lower sides, respectively, using the space-filled model. An anionic C-terminal of P16 (Asn12) is buried within the contact surface but there is no counter cationic group in the vicinity of the buried C-terminal. Asn12 is depicted by the licorice model. The yellow ribbon represents the backbone of P16. The water molecules hydrating Asn12 are depicted by the ball-and-stick model. This figure is drawn using the VMD.

There is a case where the formation of the complete electrostatic complementarity is not favorable in terms of the water entropy. In the R12:P16 complex, for example, an anionic C-terminal of P16 is buried within the contact surface but there is no counter cationic group in the vicinity of the buried C-terminal, as observed in Figure 5. Such incomplete electrostatic complementarity, which makes a positive contribution to the total-energy change upon binding (the total energy is defined as the conformational energy plus the hydration energy), is accepted when the water-entropy gain dominates, or equivalently, when the priority should be given to the water-entropy gain. The incompleteness in the electrostatic complementarity occurs in such a case, and the Apt8-hFc1 binding (16,25) mentioned in the Introduction section also provides a good example.

A hydrogen bond is usually expressed as an electrostatic attractive interaction between hydrogen and oxygen or nitrogen. The binding is accompanied by the break of solute–water hydrogen bonds compensated with the formation of solute–solute hydrogen bonds. Thus, the significance of the specific hydrogen bonding between the solute molecules upon binding can be discussed in a similar manner.

### Crucial importance of geometric characteristics of solute molecules

The entropic EV effect becomes stronger as the molecular size of solvent decreases and/or the solvent number density increases. Thanks to the hydrogen bonding, water can exist in liquid state at ambient temperature and pressure despite its exceptionally small molecular size. The effect, which is relevant to the translational displacement of solvent molecules, becomes the largest when the solvent is water. The effect of the translational, configurational entropy of water plays imperative roles in sustaining life (65–67).

**Figure 6.**
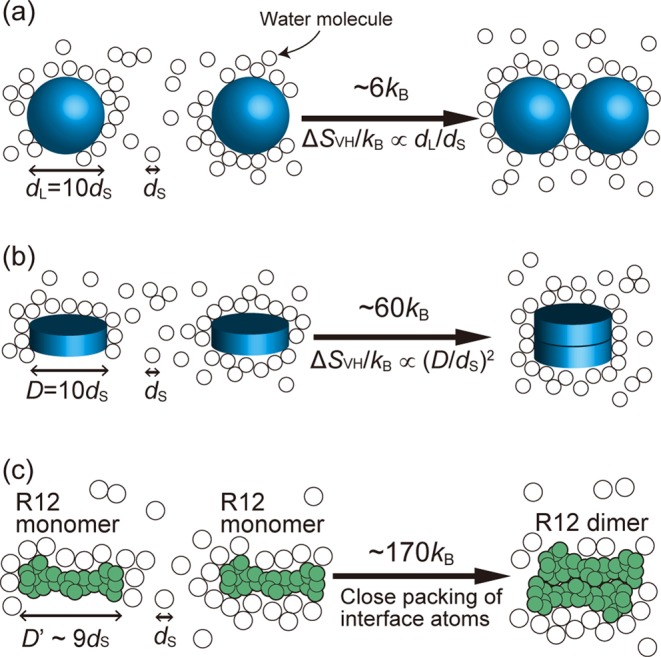
Water-entropy gain (*k*_B_ is the Boltzmann constant) upon the contact or stacking of two solutes immersed in water. (**a**) Contact of two spherical solutes with diameter *d*_L_ = 10*d*_S_ (*d*_S_ denotes the water diameter). (**b**) Stacking of two disc-shaped solutes with surface diameter *D* = 10*d*_S_. (**c**) Stacking of two R12 monomers each of which has disc-like shape with the span *D*’∼9*d*_S_. The solutes in (a) and (b) possess no polyatomic structures whereas those in (c) possess them.

When two large spherical solutes with diameter *d*_L_ contact each other as shown in Figure 6a, the water-entropy gain is ∼6*k*_B_ and increases in proportion to *d*_L_/*d*_S_ (*d*_S_ is the water diameter, 0.28 nm) (29). At *T* = 298 K, *k*_B_*T* corresponds to ∼−0.6 kcal/mol in terms of the contribution to the free-energy change. On the other hand, the water-entropy gain reaching ∼60*k*_B_ occurs upon the stacking of two large disc-shaped solutes with the surface diameter *D*, and the gain increases in proportion to (*D*/*d*_S_)^2^ (see Figure 6b) (29). It is assumed that the spherical and disc-shaped solutes possess no polyatomic structures. Thus, the water-entropy gain originating from the entropic EV effect is remarkably influenced by the overall shapes and sizes of the solutes. It is also largely dependent on the details of the solute polyatomic structure. An R12 monomer has disc-like shape with the upper-surface diameter *D*’∼9*d*_S_. The stacking of two R12 monomers to form a dimer illustrated in Figure 6c leads to the water-entropy gain of ∼170*k*_B_ that is far larger than the value expected from Figure 6b. This result arises from the close packing of the atoms constituting the interface regions. The decrease in the total EV brought by such packing is much larger than that brought by the contact of flat surfaces.

**Figure 7.**
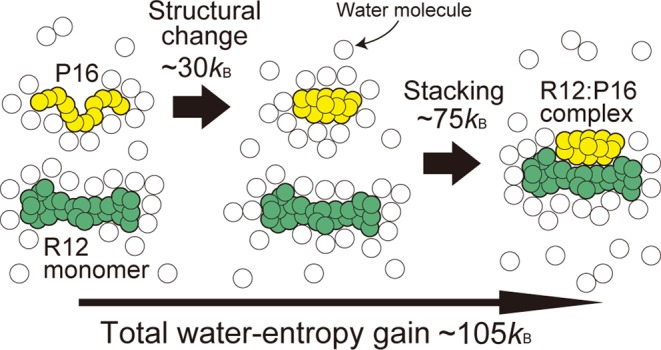
Water-entropy gain (*k*_B_ is the Boltzmann constant) upon each elemental process of the R12–P16 binding. The total water-entropy gain is also given.

The water-entropy gain upon each elemental process of the R12–P16 binding is summarized in Figure 7. Each value given is the average taken over the five models of the complex structure. The gain upon the structural change of P16 from the random-coil state to the compact one is ∼30*k*_B_. Even for the structural change of such a small (*N*_r_ = 12) polypeptide, the water-entropy gain becomes this large. The gain arising from the binding of P16 to one of the surfaces of the R12 dimer is ∼75*k*_B_. This value is roughly a half of the gain in Figure 6c, partly because P16 is smaller than R12. The total gain is ∼105*k*_B_ (105 = 30 + 75). Thus, the binding in path II is accompanied by a very large gain of the water entropy.

### Significance of stacking of flat moieties

In the R12:P16 complex, an indole ring of Trp 3 of P16 stacks on a guanidine ring of R12 (see Figure 2). This is why the π–π stacking interaction was regarded as a significant driving force for the binding (5). The π–π stacking interaction is often described by the van der Waals attractive interaction. When the stacking of flat moieties occurs, however, the van der Waals attractive interaction gained is accompanied by the loss of the moiety–water van der Waals attractive interactions, and these two factors are compensating. In other words, the stacking is required for canceling the loss. For this reason, in general, the stacking of flat moieties is often observed in a complex. The stacking is driven by a large gain of the water entropy as understood from Figure 6b or c. Looking at the structural data for a complex without accounting for the water roles can mislead to the conclusion that in the R12–P16 binding, for instance, the binding is driven by the π–π stacking interaction described above.

### Significance of shape complementarity

The entropic EV effect is responsible for the shape complementarity often observed in a complex (2). For instance, it is obvious that the lock–key binding leads to a remarkable increase in the water entropy (see Figure 3a) and the molecular recognition is attributable to the lock–key shape complementarity which provides the largest decrease in the total EV. In the induced-fit binding, the lock structure exhibits a slight structural change so as to acquire the shape complementarity. In general binding processes, the shape complementarity between portions of the interface regions surely plays essential roles, and that at the atomic level also makes a remarkably large contribution to the water-entropy increase.

### On the structural plasticity

The discussion on the significance of the electrostatic and shape complementarities made in the previous sections can also be applicable to the binding described by the lock–key or induced-fit model. We now discuss the molecular recognition accompanied by a global structural change of one of the solute molecules.

Close packing of the constituent atoms of a solute molecule leads to a large increase in the water entropy. However, such overall close packing cannot necessarily be achieved. When it is unachievable, the entropic EV effect is not large enough to surpass the energetic dehydration and conformational-entropy effects with the result that the solute molecule takes a rather extended, flexible structure. Even when the close packing can be accomplished, if the solute molecule is not sufficiently large and the energetic dehydration effect is substantial, it does not take a compact structure (68). P16 is a good example.

A protein is driven to take a structure in which the backbone and side chains are closely packed by the entropic EV effect. However, not all of the amino-acid sequences are amenable to such overall close packing. Even in cases where the overall close packing is not achievable, there are certainly the portions that can closely be packed. When preferential close packing of these portions is made, the other portions cannot participate in the packing and therefore become disordered and flexible. Nevertheless, the water-entropy gain brought by such preferential close packing is considerably larger than that resulting from an overall loose packing (69). An example case is found for PrP^C^: its N-terminal half including P1 and P16 is disordered and flexible.

P16 and PrP^C^ can be referred to as ‘a soft molecule’ and ‘a molecule possessing a soft portion’, respectively. By contrast, ‘a rigid molecule’ possesses a structure in which overall close packing of the constituent atoms is successfully achieved, and even a slight change of its structure gives rise to an unacceptably large loss of the water entropy. R12 is a good example of rigid molecules. It is possible even for a soft molecule or a soft portion of a molecule to construct a structure with overall close packing if the construction is made in concert with another molecule as a partner. In particular, when the partner is rigid, the soft molecule or the soft portion may bind to the partner by changing its structure in accordance with the partner structure, leading to the formation of a stable complex. Of course, there are not significantly many partners realizing such formation: only those which can realize it is successfully recognized.

## CONCLUSIONS

We have investigated the binding of R12 and P16 as an important example of the new type of molecular recognition accompanied by a global structural change of a molecule upon binding to its targets. In this case, P16 exhibits the global change from an unstructured state to a compact structure while the structure of R12 remains almost unchanged (5,23). Changes in thermodynamic quantities upon binding are calculated using the molecular mechanics, hybrid method (30–37) in which the ADIET (38–42) is combined with the MA (43,44), and 3D-RISM theory (45–48). Molecular models are employed for water. The hybrid method and the 3D-RISM theory have been quite successful in studies on a variety of biological self-assembly processes (27–32,49–52) such as protein folding, association of protein molecules and receptor–ligand binding. In the present study, the hybrid method and the 3D-RISM theory are employed in calculating the hydration entropy and energy, respectively. This employment provides the most reliable results even in a quantitative sense.

The energy decrease due to the gain of R12–P16 attractive (i.e. van der Waals and electrostatic) interactions is almost canceled out by the energy increase originating from the energetic dehydration effect (i.e. by the energy increase caused by the loss of R12–water and P16–water attractive interactions plus the energy lowering arising from the structural reorganization of water). The binding is driven by the water-entropy gain that predominates over the conformational-entropy loss upon the global structural change of P16. The water-entropy gain originates primarily from the overlap of EVs of R12 and P16 followed by the increase in the total volume available to the translational displacement of water molecules in the system by the overlapped volume. Here, the EV is the volume of the space which the centers of water molecules cannot enter. The binding gives rise to an energy decrease arising from van der Waals attractive interactions between R12 and P16, but it is accompanied by an energy increase related to the loss of R12–water and P16–water van der Waals attractive interactions: the former is slightly larger and the net change (net change 1) is negative. The binding brings an energy decrease arising from electrostatic attractive interactions between R12 and P16, but it is accompanied by an energy increase related to the loss of R12–water and P16–water electrostatic attractive interactions; the latter is slightly larger and the net change (net change 2) is positive. The loss of R12–water and P16–water attractive interactions is quite large. However, about half of it is canceled out by the water reorganization energy (26,27), energy lowering due to the structural reorganization of water upon binding. The resultant loss becomes comparable with the gain of R12–P16 attractive interactions. Net changes 1 and 2 are also compensating.

The picture of the binding can be summarized as follows. The binding is driven by the water-entropy gain which overcomes the dehydrations of R12 and P16 with the aid of a gain of R12–P16 van der Waals and electrostatic attractive interactions. A loss of the conformational entropy of P16, which opposes the binding, is surpassed by the water-entropy gain. It is very important to assure the gain of R12–P16 attractive interactions during the binding process though the gain itself is not a driving force. We believe that this picture is applicable to general binding processes. This is why stacking of flat moieties, specific hydrogen bonding and electrostatic complementarity (i.e. contact of oppositely charged groups) are frequently observed in the complexes (2). Stacking of flat moieties leads to a gain of intermolecular van der Waals attractive interactions, and specific hydrogen bonding and electrostatic complementarity lead to gains of intermolecular electrostatic attractive interactions. However, there is a case where it is not possible to accomplish both the water-entropy gain and the electrostatic complementarity. When the water-entropy gain dominates, or equivalently, when the priority should be given to the water-entropy gain, the electrostatic complementarity becomes incomplete. Such incompleteness has been found in the Apt8-hFc1 binding (16,25).

The geometric characteristics (overall shapes, sizes and details of the polyatomic structures) of the solute molecules play crucially important roles in discussing the water-entropy gain. For instance, stacking of disc-shaped solutes brings a large decrease in the total EV followed by a correspondingly large gain of the water entropy. The gain becomes larger as the solute size increases (29). These effects are enlarged when the interface atoms are closely packed. The reason why stacking of flat moieties is frequently observed in the complexes (2) can also be understood from this viewpoint. Further, it is obvious that the water-entropy gain becomes quite large when the shape complementarity occurs within the interface region. The most striking example is found in the lock–key binding (see Figure 3a). The induced-fit binding, in which the lock structure exhibits a slight structural change so as to acquire the shape complementarity, is also driven by a large gain of the water entropy.

In what follows, we describe our proposition concerning the mechanism of molecular recognition accompanied by a global structural change of a molecule upon binding to its targets. When a solute molecule is not sufficiently large, the water-entropy gain is often incapable of surpassing the energetic dehydration and conformational-entropy effects with the result that the solute molecule takes a rather extended, flexible structure (68). Even for a large solute molecule like a protein, when its overall close packing is not achievable, only the portions that can closely be packed are preferentially packed: the other portions cannot participate in the close packing and therefore become disordered and flexible (69). (The water-entropy gain brought by such preferential close packing is considerably larger than that resulting from an overall loose packing.) These solute molecules can be referred to as ‘a soft molecule’ and ‘a molecule possessing a soft portion’, respectively. By contrast, a solute molecule possessing a structure in which overall close packing of the constituent atoms is successfully achieved is ‘rigid’ because even a slight change of its structure gives rise to an unacceptably significant loss of the water entropy. It is possible even for a soft molecule or a soft portion of a molecule to construct a structure with overall close packing if the construction is made in concert with another molecule as a partner. In particular, when the partner is rigid, the soft molecule or the soft portion may bind to the partner by changing its structure in accordance with the partner structure, leading to the formation of a stable complex. Of course, there are not significantly many partners realizing such formation: only those which can realize it is successfully recognized.

Last, it is worthwhile to recapitulate the mechanism of the R12–P16 binding. For the binding, P16 changes its structure so that the R12–P16 interface atoms can closely be packed. At the same time, however, a decrease in R12–P16 intermolecular energy as well as that in P16 intramolecular energy (i.e. a gain of van der Waals and electrostatic attractive interactions between R12 and P16 and that within P16) is required to compensate for the energetic dehydration effect. As for the R12–P16 intermolecular energy, the formations of stacking structure and of electrostatic complementarity lead to decreases in van der Waals and electrostatic interaction energies, respectively. Since the stacking of flat moieties and the contact of unlike-charged groups bring decreases in the EVs, the formations are also favorable in terms of the water-entropy gain. The water-entropy gain brought by close R12–P16 interface packing, with the help of the decrease in the sum of P16 intramolecular and R12–P16 intermolecular energies, is large enough to surpass the energetic dehydration effect and the conformational-entropy loss of P16: the R12–P16 binding is achieved. If it was not large enough, the binding and the molecular recognition would not be realized. For the energetic component, the decrease in the sum of P16 intramolecular and R12–P16 energies, which is in favor of the binding, is considerably smaller than the opposing energetic dehydration effect. For the entropic component, by contrast, the water-entropy gain promoting the binding is far larger than the opposing conformational-entropy loss of P16.

To demonstrate our proposition mentioned in the last paragraph but one, we intend to investigate the binding of another RNA aptamer to its targets upon which the RNA aptamer exhibits a global structural change. The binding of an intrinsically disordered protein to its targets is also an interesting subject to be tackled. The ultimate goal is to elucidate all types of molecular recognitions including the ‘lock and key’ and ‘induced-fit‘ binding models within the same theoretical framework in a unified manner.

## ACCESSION NUMBER

The coordinates of the ensemble of five structures for the 2×R12:2×P16 complex have been deposited in the Protein Data Bank with the accession code 2RU7.

## SUPPLEMENTARY DATA

Supplementary Data are available at NAR Online, including [1–18].

SUPPLEMENTARY DATA
