# Supplementary material for: Binding of an RNA aptamer and a partial peptide of a prion protein: crucial importance of water entropy in molecular recognition
Source: Nucleic Acids Res. 2014 May 6;42(11):6861–75. doi: 10.1093/nar/gku382 (PMC4066790; doi:10.1093/nar/gku382)
Supplement: SUPPLEMENTARY DATA [file supp_42_11_6861__index.html]

SUPPLEMENTARY DATA 

# Binding of an RNA aptamer and a partial peptide of a prion protein: crucial importance of water entropy in molecular recognition

## SUPPLEMENTARY DATA

**Files in this Data Supplement:**

- Supplementary Data
