# Supplementary material for: Binding of an RNA aptamer and a partial peptide of a prion protein: crucial importance of water entropy in molecular recognition
Source: Nucleic Acids Res. 2014 May 6;42(11):6861–75. doi: 10.1093/nar/gku382 (PMC4066790; doi:10.1093/nar/gku382)
Supplement: SUPPLEMENTARY DATA [file supp_gku382_nar-00374-r-2014-File009.pdf]

Supplementary Appendices

Binding of an RNA Aptamer and a Partial Peptide of a Prion Protein: Crucial Importance of Water Entropy in Molecular Recognition

Tomohiko Hayashi, Hiraku Oshima, Tsukasa Mashima, Takashi Nagata, Masato Katahira, and Masahiro Kinoshita*

Institute of Advanced Energy, Kyoto University, Uji, Kyoto 611-0011, Japan

**To whom correspondence should be addressed at e-mail: kinoshit@iae.kyoto-u.ac.jp*

Table of Contents

A. Calculation of Dissociation Constant for 2×R12:2×P16 Complex S2

B. Calculation of Hydration Entropy by the Angle-Dependent Integral Equation Theory S7

C. Calculation of Hydration Energy by the Three-Dimensional Reference Interaction Site Model Theory S11

References S14

A. Calculation of Dissociation Constant for 2×R12:2×P16 Complex

The binding of a P16 to an R12 monomer was assumed in a previous work (1). The dissociation constant K_D was then calculated for the R12:P16 complex by considering the binding reaction,

$$\mathrm{R12{\cdot}P16} \overset{K_D}{\Leftrightarrow} \mathrm{R12+P16}.$$

However, R12 is present as a dimer and two P16s bind to the R12 molecules forming the dimer, respectively (see Figure 1 in the main manuscript) (1). Therefore, the binding reaction should be expressed as follows:

$$\mathrm{R12^2{\cdot}P16} \overset{K_{D1}}{\Leftrightarrow} \mathrm{R12^2+P16},$$

$$\mathrm{R12^2{\cdot}P16^2} \overset{K_{D2}}{\Leftrightarrow} \mathrm{R12^2{\cdot}P16+P16}.$$

Here, the superscript "2" represents a dimer of R12 or two P16s bound, and K_{D1} and K_{D2} are the *macroscopic* dissociation constants for the two macroscopic equilibria, respectively. The method of calculating the dissociation constant for the 2×R12:2×P16 complex ($R12^2$:$P16^2$) is different from that for the R12:P16 complex (R12:P16).

In the filter binding assay, aqueous solution of R12 dimer and P16 is flowed through nitrocellulose membrane filters. R12 dimers with no P16s bound pass freely through the filter, whereas P16s and R12 dimers to which a P16 is or two P16s are bound are retained. RNAs are labeled by a radioactive tag, and the total amount of 2×R12:P16 ($R12^2$:P16) and 2×R12:2×P16 ($R12^2$:$P16^2$) complexes retained can be measured by radiation counters. We assume that the 2×R12:P16 and 2×R12:2×P16 complexes are retained at the same rate. K_{D1} and K_{D2} are defined by

$$K_{D1} = \frac{[R12^2][P16]}{[R12^2 \cdot P16]}, \tag{S1}$$

$$K_{D2} = \frac{[R12^2 \cdot P16][P16]}{[R12^2 \cdot P16^2]}. \tag{S2}$$

In the filter binding assay, the binding activity is measured as "the total amount of R12 dimer to which a P16 is or two P16s are bound" divided by "the total amount of R12 dimer",

$$\xi = \frac{[R12^2 \cdot P16] + [R12^2 \cdot P16^2]}{[R12^2]_0}, \tag{S3}$$

where $[R12^2]_0 = [R12^2] + [R12^2 \cdot P16] + [R12^2 \cdot P16^2]$. Substituting Eqs. S1 and S2 into Eq. S3 yields

$$\xi = \frac{K_{D2}[P16] + [P16]^2}{K_{D1}K_{D2} + K_{D2}[P16] + [P16]^2}. \tag{S4}$$

Since an R12 dimer has two binding sites, which are hereafter referred to as binding sites "a" and "b", respectively, the 2×R12:1×P16 complex can be expressed as a mixture of two distinct components as

$$[R12^2 \cdot P16] = [R12^2 \cdot P16_a] + [R12^2 \cdot P16_b] \tag{S5}$$

where $[R12^2 \cdot P16_a]$ and $[R12^2 \cdot P16_b]$ are the mole concentrations of the 2×R12:P16 complex in which P16s are bound to binding sites "a" and "b" of the R12 dimer, respectively. The four *microscopic* equilibria are then defined using the microscopic dissociation constants by

$$k_{D1} = \frac{[R12^2][P16]}{[R12^2 \cdot P16_a]}, \tag{S6}$$

$$k_{D2} = \frac{[R12^2][P16]}{[R12^2 \cdot P16_b]}, \tag{S7}$$

$$k_{D3} = \frac{[R12^2 \cdot P16_a][P16]}{[R12^2 \cdot P16^2]}, \tag{S8}$$

$$k_{D4} = \frac{[R12^2 \cdot P16_b][P16]}{[R12^2 \cdot P16^2]}. \tag{S9}$$

From Eqs. S1, S2, and S5 to S9, we obtain the relationship between the macroscopic and microscopic dissociation constants as

$$K_{D1} = \frac{1}{\frac{1}{k_{D1}} + \frac{1}{k_{D2}}}, \tag{S10}$$

$$K_{D2} = 2\frac{\frac{1}{k_{D1}} + \frac{1}{k_{D2}}}{\frac{1}{k_{D1}k_{D3}} + \frac{1}{k_{D2}k_{D4}}}. \tag{S11}$$

In the 2×R12:2×P16 complex, the two P16s are well separated (i.e., bound to the opposite sides of the R12 dimer) and the R12 dimer is rigid. Therefore, it can reasonably be assumed that the binding of P16 to one side has no effect on that to the other side. That is, the four subreactions occur independently. We further assume that the four microscopic constants k_{Di} (i = 1, 2, 3, 4) take the same value denoted by k_D. The macroscopic dissociation constants K_{D1} and K_{D2} can be rewritten using k_D as

$$K_{D1} = k_D/2, \tag{S12}$$

$$K_{D2} = 2k_D. \tag{S13}$$

Eq. S4 then becomes

$$\xi = \frac{2k_D[P16] + [P16]^2}{(k_D+[P16])^2}. \tag{S14}$$

The concentration of P16 unbound to the R12 dimer, [P16], can be approximated by the total concentration of P16, $[P16]_0$. This is because only a very small percentage of P16 is bound to the dimer in the experiment where ξ is obtained as a function of $[P16]_0$. The parameter k_D is determined by fitting $\xi=B_{max}(2k_D[P16]_0+[P16]_0^2)/(k_D+[P16]_0)^2$ (B_{max} is the maximum value at the saturating P16 concentration) to the experimentally obtained curve in terms of B_{max} and k_D. The fitting is performed using the nonlinear least square method. Figure S1 shows that the results of the fitting: k_D = 18.71±5.64 μM and B_{max} = 17.17±1.51. The binding free energy ΔG is defined by

$$\Delta G = -RT\ln k_D. \tag{S15}$$

Here, R is the gas constant and T is the absolute temperature. At T = 298.15 K, we obtain ΔG = −6.45 kcal/mol. This can be regarded as the free-energy change upon the binding of a P16 to a R12 monomer considered in the present study.

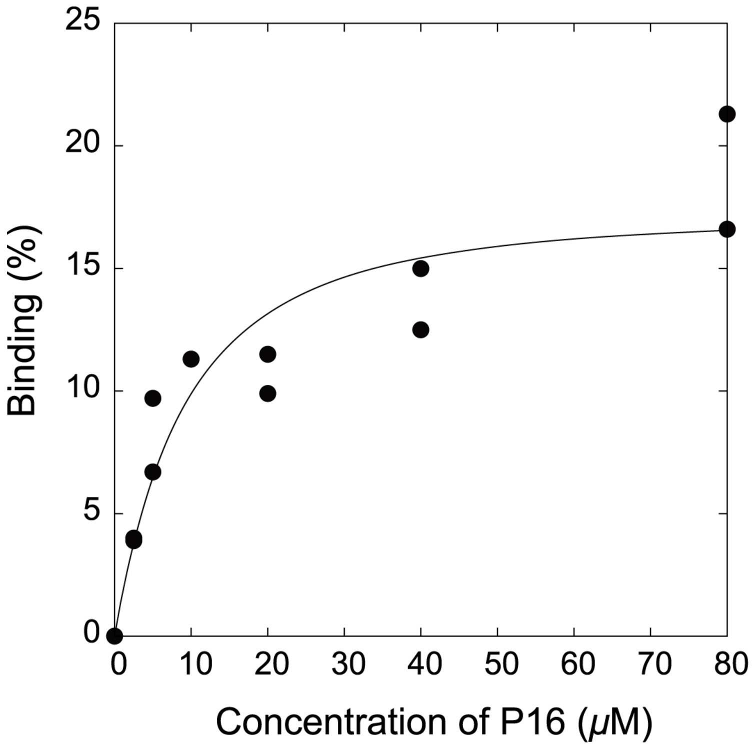

Figure S1. Saturation binding curve for complexes of R12 dimer and P16. "Binding (%)" represents 100ξ where ξ represents "the total amount of R12 dimer to which a P16 is or two P16s are bound" divided by "the total amount of R12 dimer". Black circles represent the experimental data (1).

B. Calculation of Hydration Entropy by the Angle-Dependent Integral Equation Theory

A water molecule is modeled as a hard sphere with diameter d_S = 0.28 nm in which a point dipole and a point quadrupole of tetrahedral symmetry are embedded (2,3). The absolute temperature T is set at 298 K and the water number density ρ_S is taken to be that of real water: ρ_S = 0.0333 Å^{-3}. The values of $\rho_S d_S^3$ is 0.7317. The influence of molecular polarizability of water is taken into account by employing the self-consistent mean field (SCMF) theory (2,3). At the SCMF level, the many-body induced interactions are reduced to pairwise additive potentials involving an effective dipole moment. The effective dipole moment thus determined is about 1.42 times larger than the bare gas-phase dipole moment. The dimensionless dipole and quadrupole moments, $\mu_S^* = \mu_S/(d_S^3 k_B T)^{1/2}$ and $\theta_S^* = \theta_S/(d_S^5 k_B T)^{1/2}$ (k_B is the Boltzmann constant), are 2.768 and 0.968, respectively.

A spherical solute is considered in the analysis using the angle-dependent integral equation theory (ADIET) (2–7). Hard spheres of diameter d_U (i.e., solutes) are immersed in the model water. The solute-water interaction potential (u_{US}) is expressed as

$$u_{US}(r) = \infty \qquad \text{for } r < (d_U + d_S)/2, \tag{S16a}$$

$$u_{US}(r) = 0 \qquad \text{for } r \geq (d_U + d_S)/2, \tag{S16b}$$

where r is the distance between the centers of a solute and a water molecule.

The ADIET is briefly described here. The Ornstein-Zernike (OZ) equation can be written as:

$$\eta_{\alpha\beta}(12) = \frac{1}{8\pi^2}\sum_{\gamma}\rho_\gamma \int c_{\alpha\gamma}(13)\left\{\eta_{\gamma\beta}(32) + c_{\gamma\beta}(32)\right\} d3, \tag{S17a}$$

$$\eta_{\alpha\beta}(12) = h_{\alpha\beta}(12) - c_{\alpha\beta}(12); \alpha, \beta = \mathrm{S}, \mathrm{U}, \tag{S17b}$$

where the subscripts S and U denote solvent (water) and solute, respectively, h and c are the total and direct correlation functions, respectively, (ij) represents ($\mathbf{r}_{ij}$, $\mathbf{\Omega}_i$, $\mathbf{\Omega}_j$), $\mathbf{r}_{ij}$ is the vector connecting the center of particles i and j, $\mathbf{\Omega}_i$ denotes the three Euler angles describing the orientation of particle i, and $\int d3$ represents integration over all position and angular coordinates of particle 3. The closure equation is expressed by (2–7)

$$c_{\alpha\beta}(12) = \int_{r_{12}}^{\infty} \left[h_{\alpha\beta}(12) \frac{\partial \{ w_{\alpha\beta}(12) - b_{\alpha\beta}(12) \}}{\partial r'_{12}} \right] dr'_{12} - \frac{u_{\alpha\beta}(12)}{k_{\mathrm{B}}T} + b_{\alpha\beta}(12), \tag{S18a}$$

$$w_{\alpha\beta}(12) = -\eta_{\alpha\beta}(12) + \frac{u_{\alpha\beta}(12)}{k_{\mathrm{B}}T}, \tag{S18b}$$

where u is the pair potential, b is the bridge function, and r_{12} is the distance between the centers of two particles. In the present analysis, the hypernetted-chain (HNC) approximation is employed (b = 0). We assume that the solutes are immersed in water at infinite dilution (ρ_{U} = 0). The water-water and solute-water interaction potentials, T, and ρ_{S} form the input data. The calculation process can then be split into two steps:

1. Solve Eqs. S17 and S18 for pure (bulk) water. Calculate the correlation functions X_{SS} ($X = h, c$).
2. Solve Eqs. S17 and S18 for water in which a solute is immersed using the correlation functions obtained in step 1 as part of the input data. Calculate the correlation functions X_{US} ($X = h, c$).

For the numerical solution of Eqs. S17 and S18, the pair potentials and correlation functions are expanded in a basis set of rotational invariants, and the basic equations are reformulated in terms of the projections $X_{\mu\nu}^{mnl}(r)$ occurring in the rotational-invariant expansion of X (2–7). The expansion considered for m, $n \leq n_{\max} = 4$ gives sufficiently accurate results for hard-sphere solutes. The basic equations are then numerically solved using the robust, highly efficient algorithm developed by Kinoshita and coworkers (4,5). In the numerical treatment, a sufficiently long range r_L is divided into N grid points ($r_i = i\delta r$, $i = 0, 1, \ldots, N-1$; $\delta r = r_L/N$) and all of the projections are represented by their values on these points. The grid width and the number of grid points are set at $\delta r = 0.01d_S$ and $N =$ 4096, respectively.

The hydration free energy μ_H is calculated using the Morita-Hiroike formula extended to molecular liquids (8,9) as

$$\frac{\mu_H}{k_B T} = \frac{\rho_S}{8\pi^2} \int\int\int\int 4\pi \left[\frac{1}{2}\{h_{US}(r,\theta,\phi,\chi)\}^2 - \frac{1}{2} h_{US}(r,\theta,\phi,\chi) c_{US}(r,\theta,\phi,\chi) - c_{US}(r,\theta,\phi,\chi)\right] r^2 \sin\theta \, dr d\theta d\phi d\chi, \qquad \text{(S19)}$$

where the integration range is $[0, \infty]$ for r, $[0, \pi]$ for θ, and $[0, 2\pi]$ for ϕ and χ. The hydration entropy S_{VH} is evaluated through the numerical differentiation of μ_H with respect to the temperature at constant density as (5)

$$S_{VH} = -\left(\frac{\partial \mu_H}{\partial T}\right)_{\rho_S} \cong -\frac{\mu_H(T + \Delta T) - \mu_H(T - \Delta T)}{2\Delta T}, \ \Delta T = 5\text{K}. \qquad \text{(S20)}$$

The quantitative reliability of the angle-dependent integral equation theory combined with the

multipolar water model in calculating μ_H has been demonstrated by comparing the results for nonpolar solutes with those from computer simulation studies, as described in the main manuscript (MODEL AND THEORY).

C. Calculation of Hydration Energy by the Three-Dimensional Reference Interaction Site Model Theory

Consider a system composed of one solute and one solvent molecular species (water in this study). A solvent molecule consists of N^{V} atomic sites. The solvent sites are numbered consecutively, $\gamma = 1, \ldots, N^{\mathrm{V}}$ (for a water molecule, the number of solvent sites is 3; an oxygen and two hydrogens). For this system, the three-dimensional (3D) reference interaction site model (RISM) equation (10–13) is written as

$$h_\gamma(\mathbf{r}) = \sum_{\gamma'} c_{\gamma'}(\mathbf{r}) * \left\{ w^{vv}_{\gamma'\gamma}(r) + \rho_{\mathrm{S}} h^{vv}_{\gamma'\gamma}(r) \right\}, \qquad \text{(S21)}$$

where $h_\gamma(\mathbf{r})$ and $c_\gamma(\mathbf{r})$ are the 3D total and direct correlation functions between solvent site γ and the solute, respectively, the asterisk denotes a convolution integral in the real space, $w^{vv}_{\gamma'\gamma}(r)$ is the site-site intramolecular correlation function representing the molecular structure of the solvent, ρ_{S} is the solvent number density. The site-site total correlation function for bulk solvent, $h^{vv}_{\gamma'\gamma}(r)$, is calculated in advance using the dielectrically consistent RISM (DRISM) theory (14,15). The 3D-RISM equation is complemented by the 3D version of the Kovalenko-Hirata (KH) closure approximation (11) as

$$\begin{aligned} g_\gamma(\mathbf{r}) &= \begin{cases} \exp\{d_\gamma(\mathbf{r})\} & \text{for } d_\gamma(\mathbf{r}) \le 0, \\ 1 + d_\gamma(\mathbf{r}) & \text{for } d_\gamma(\mathbf{r}) > 0, \end{cases} \\ d_\gamma(\mathbf{r}) &= -\frac{u_\gamma(\mathbf{r})}{k_{\mathrm{B}}T} + h_\gamma(\mathbf{r}) - c_\gamma(\mathbf{r}), \end{aligned} \qquad \text{(S22)}$$

where $g_\gamma(\mathbf{r}) = h_\gamma(\mathbf{r}) + 1$ is the 3D distribution function of solvent site γ around the solute. (The KH closure is also employed for bulk solvent.) The function $u_\gamma(\mathbf{r})$ is the 3D interaction potential

between solvent site γ and the solute, which is calculated on the supercell grid using the minimum image convention and the Ewald summation.

The calculation procedure is as follows. First, the site-site correlation functions for bulk water are calculated using the DRISM theory (14,15) coupled with the KH closure equation (11). The site-site intermolecular potentials, T (= 298 K), and ρ_S (= 0.0333 Å^{-3}) are served as the input data. The extended single point charge (SPC/E) model (16) is employed for water with a correction in terms of the LJ potential parameters for the hydrogen sites (σ = 0.654 Å, ε = 0.0155 kcal/mol). The LJ potential parameters and partial charges for the solute atoms are taken from the standard Amber99SB force field (17). The water-solute correlation functions are then obtained for the solute molecule with a prescribed structure by solving the 3D-RISM/KH equations. The site-site correlation functions for bulk water and the water-solute interaction potentials form the input data. The solution is performed on a 3D cubic grid. The grid spacing (Δx, Δy, and Δz) is set at 0.5 Å, and the grid resolution ($N_x \times N_y \times N_z$) is 256×256×256. It has been confirmed that the spacing is sufficiently small and the box size ($N_x\Delta x$, $N_y\Delta y$, $N_z\Delta$z) is large enough for the calculation result to become identical within convergence tolerance.

The hydration free energy μ_H is calculated using the 3D extension of Singer-Chandler formula adapted to the KH closure equation as (11,18)

$$\frac{\mu_H}{k_B T} = \rho_S \sum_{\gamma} \int d\mathbf{r} \left[\frac{1}{2}\{h_\gamma(\mathbf{r})\}^2 \Theta(-h_\gamma(\mathbf{r})) - c_\gamma(\mathbf{r}) - \frac{1}{2} h_\gamma(\mathbf{r}) c_\gamma(\mathbf{r})\right], \tag{S23}$$

where Θ is the Heaviside function. The hydration entropy $S_{\rm VH}$ is evaluated through the numerical differentiation of $\mu_{\rm H}$ with respect to T at constant $\rho_{\rm S}$ (see Eq. S20). The solvation energy $\varepsilon_{\rm VH}$ is calculated from

$$\varepsilon_{\rm VH} = \mu_{\rm VH} + TS_{\rm VH}. \tag{S24}$$

Furthermore, we decompose $\varepsilon_{\rm VH}$ as

$$\varepsilon_{\rm VH} = \varepsilon_{\rm VH,\,LJ} + \varepsilon_{\rm VH,\,ES}, \tag{S25}$$

where $\varepsilon_{\rm VH,\,LJ}$ and $\varepsilon_{\rm VH,\,ES}$ are the non-electrostatic and electrostatic contributions to $\varepsilon_{\rm VH}$, respectively. The decomposition is made in the following manner. First, we calculate the hydration energy of a hypothetical solute molecule whose partial charges are all switched to zero, $\varepsilon_{\rm VH,\,LJ}$. Then, we obtain the electrostatic contribution, $\varepsilon_{\rm VH,\,ES}$, from $\varepsilon_{\rm VH,\,ES} = \varepsilon_{\rm VH} - \varepsilon_{\rm VH,\,LJ}$.

References

(1) Mashima,T., Nishikawa,F., Kamatari,Y.O., Fujiwara,H., Saimura,M., Nagata,T., Kodaki,T., Nishikawa,S., Kuwata,K. and Katahira,M. (2013) Anti-prion activity of an RNA aptamer and its structural basis. *Nucleic Acids Res.*, **41**, 1355–1362.

(2) Kusalik,P.G. and Patey,G.N. (1988) On the molecular theory of aqueous electrolyte solutions. I. The solution of the RHNC approximation for models at finite concentration. *J. Chem. Phys.*, **88**, 7715–7738.

(3) Kusalik,P.G. and Patey,G.N. (1988) The solution of the reference hypernetted-chain approximation for water-like models. *Mol. Phys.*, **65**, 1105–1119.

(4) Kinoshita,M., Harada,M. (1991) Numerical solution of the HNC equation for fluids of non-spherical particles. An efficient method with application to dipolar hard spheres. *Mol. Phys.*, **74**, 443–464.

(5) Kinoshita,M. and Bérard,D.R. (1996) Analysis of the bulk and surface-induced structure of electrolyte solutions using integral equation theories. *J. Comput. Phys.*, **124**, 230–241.

(6) Kinoshita,M. (2008) Molecular origin of the hydrophobic effect: analysis using the angle-dependent integral equation theory. *J. Chem. Phys.*, **128**, 024507.

(7) Kinoshita,M. and Yoshidome,T. (2009) Molecular origin of the negative heat capacity of hydrophilic hydration. *J. Chem. Phys.*, **130**, 144705.

(8) Morita,T. (1960) Theory of classical fluids: hyper-netted chain approximation. III. *Prog. Theor. Phys.*, **23**, 829–845.

(9) Morita,T. and Hiroike,K. (1961) A new approach to the theory of classical fluids. III. *Prog. Theor. Phys.*, **25**, 537–578.

(10) Beglov,D. and Roux,B. (1995) Numerical solution of the hypernetted chain equation for a solute of arbitrary geometry in three dimensions. *J. Chem. Phys.*, **1995**, 103, 360–364.

(11) Kovalenko,A. and Hirata,F. (1999) Self-consistent description of a metal–water interface by the Kohn–Sham density functional theory and the three-dimensional reference interaction site model. *J. Chem. Phys.*, **110**, 10095–10112.

(12) Imai,T., Harano,Y., Kinoshita,M., Kovalenko,A. and Hirata,F. (2006) A theoretical analysis on hydration thermodynamics of proteins. *J. Chem. Phys.*, **125**, 024911.

(13) Luchko,T., Gusarov,S., Roe,D.R., Simmerling,C., Case,D.A., Tuszynski,J. and Kovalenko,A. (2010) Three-dimensional molecular theory of solvation coupled with molecular dynamics in Amber. *J. Chem. Theory Comput.*, **6**, 607–624.

(14) Perkyns,J.S. and Pettitt,B.M. (1992) A dielectrically consistent interaction site theory for solvent–electrolyte mixtures. *Chem. Phys. Lett.*, **190**, 626–630.

(15) Perkyns,J.S. and Pettitt,B.M. (1992) A site–site theory for finite concentration saline solutions. *J. Chem. Phys.*, **97**, 7656–7666.

(16) Berendsen,H.J.C., Grigera,J.R. and Straatsma,T.P. (1987) The missing term in effective pair potentials. *J. Phys. Chem.*, **91**, 6269–6271.

(17) Hornak,V., Abel,R., Okur,A., Strockbine,B., Roitberg,A. and Simmerling,C. (2006) Comparison of multiple Amber force fields and development of improved protein backbone parameters. *Proteins*, **65**, 712–725.

(18) Singer,S.J. and Chandler,D. (1985) Free energy functions in the extended RISM approximation. *Mol. Phys.*, **55**, 621–625.
